# Morphological, Ultrastructural, and Molecular Aspects of In Vitro Mouse Embryo Implantation on Human Endometrial Mesenchymal Stromal Cells in The Presence of Steroid Hormones as An Implantation Model

**DOI:** 10.22074/cellj.2018.5221

**Published:** 2018-05-28

**Authors:** Marzieh Rahimipour, Mojdeh Salehnia, Mina Jafarabadi

**Affiliations:** 1 Department of Anatomy, Faculty of Medical Sciences, Tarbiat Modares University, Tehran, Iran; 2 Reproductive Health Research Center, Tehran University of Medical Sciences, Tehran, Iran

**Keywords:** Estrogen, Implantation, Interleukin-1 Receptor, Mesenchymal Stromal Cells, Progesterone

## Abstract

**Objective:**

This experimental study aimed to evaluate the effects of 17β-estradiol (E2) and progesterone (P4) on the interaction between mouse embryo and human endometrial mesenchymal stromal cells, and gene expressions related to implantation [αV and *β3* integrins, interleukin-1 receptor (*IL-1R*), and leukemia inhibitory factor receptor (LIFR)] using an *in vitro* two- dimensional model.

**Materials and Methods:**

In this experimental study, the endometrial stromal cells were isolated enzymatically and mechanically, and cultured to the fourth passage. Next, their immunophenotype was confirmed by flow cytometric analysis as mesenchymal stromal cells. The cells were cultured as either the experimental group in the presence of E2 (0.3 nmol) and P4 (63.5 nmol) or control group without any hormone treatment. Mouse blastocysts were co-cultured with endometrial mesenchymal stromal cells in both groups for 48 hours. Their interaction was assessed under an inverted microscope and scanning electron microscopy (SEM). Expressions of *αV* and *β3* integrins, *LIFR*, and *IL-1R* genes were analyzed by real-time reverse transcription-polymerase chain reaction (RT-PCR).

**Results:**

Similar observations were seen in both groups by light microscopy and SEM. We observed the presence of pinopode-like structures and cell secretions on the apical surfaces of endometrial mesenchymal stromal cells in both groups. The trophoblastic cells expanded and interacted with the mesenchymal monolayer cells. At the molecular level, expression of *IL-1R* significantly increased in the hormonal treated group compared to the control (P≤0.05). Expressions of the other genes did not differ.

**Conclusion:**

This study has shown that co-culture of endometrial mesenchymal stromal cells with mouse embryo in media that contained E2 (0.3 nmol) and P4 (63.5 nmol) could effectively increase the expression of *IL-1R*, which is involved in embryo implantation. However, there were no significant effects on expressions of *αV* and *β3* integrins, *LIFR*, and on the morphology and ultrastructure of endometrial mesenchymal stromal cells.

## Introduction

Implantation is a complex process that involves fine coordination and dialogue between the embryo and endometrium (1). Embryonic development to the blastocyst stage and uterine differentiation to the receptive phase are both essential for initiation and progression of a successful implantation (2). The process of implantation consists of apposition, adhesion, and the invasion of the blastocyst to the uterine wall (3).

In addition to the physical interaction between the embryo and uterine cells, this process is influenced by maternal steroidal hormones, growth factors, and cytokines in a paracrine manner that play a vital role in embryonic signaling (4). Uterine differentiation to support embryo implantation is coordinated by progesterone (P4) and 17ß-estradiol (E2) (5, 6). In mice and rats both maternal P4 and E2 are critical to implantation. However, in most species such as hamsters, rabbits, and pigs, implantation can occur in the presence of P4 alone (7). The implantation process involves different factors and proteins such as leukemia inhibitory factor (*LIF*) (3), interleukin-1 (*IL-1*), interleukin-1 receptor (*IL-1R*) (8), and integrins (9). 

The highest level of *LIF* in the endometrial epithelium is expressed during the implantation window (3). The embryo is also capable of regulating endometrial production of *LIF* (10). Pre-implantation embryos (11) and cytotrophoblasts (12) express *LIF* and its receptor (*LIFR*). *LIF* promotes endometrial receptivity and increases the adhesion of trophoblastic cells to endometrial cells by upregulating expression of *αVß3* and *αVß5* (13).

*IL-1* has several functions in the window of implantation. It stimulates endometrial secretion of *LIF*, prostaglandin E2, and integrin *ß3* subunit expression (8, 14). Research indicates that *IL-1* and *IL-1R1* are expressed by blastocysts. In early pregnancy, *IL-1R1* is predominantly expressed in syncytiotrophoblasts and endometrial glands. Its mRNA is upregulated during decidualization of endometrial stromal cells *in vitro* (15). 

Integrins are a family of transmembrane glycoproteins with two subunits, a and ß. They act as receptors for extracellular matrix components and other cells (16). Integrin expressions increase in the phase of receptivity of the endometrium and are considered markers of the implantation window (9). The cycle-specific expression patterns of endometrial integrins indicate their hormonal regulation (17). These proteins are expressed on the endometrium and the blastocyst. The human blastocyst expresses *αVß3* as well as *a3ß1, a6ß4,* and *αVß5* (18, 19).

Ethical restrictions and experimental limitations prevent direct evaluation of interactions between the embryo and endometrium at the morphological and molecular levels. So, the application of *in vitro* implantation models could be useful to gain better knowledge about the implantation process and to evaluate the effects of different factors involved in implantation. Until now, several *in vitro* implantation models have been introduced by different groups using two- and three-dimensional culture systems. Several studies separately used endometrial epithelial or stromal cells, whereas others used the combination of stromal and epithelial cells to establish implantation models (20). The implantation models could be a valuable alternative tool for more investigations regarding the mechanism of implantation. 

Our previous studies demonstrated that passage-4 endometrial mesenchymal stromal cells expressed typical markers of mesenchymal stromal stem cells. They could differentiate into different cell lines (21, 22). According to our knowledge, there is scant information about the establishment of implantation models using endometrial stromal cells. Recently, Fayazi et al. (23) showed that the CD146^+^ endometrial mesenchymal cells could differentiate to endometrial epithelial-like cells. However, in this study, the researchers did not evaluate the interaction of these epithelial-like cells with embryos. 

Ovarian hormones have critical roles during embryo implantation. These hormones regulate the specific gene products that may play important roles in embryo implantation (24). The profile of genes expression in rodents and human endometrium using *in vivo* administration of E2 has been shown by several investigators (25). In these *in vivo* experiments the studied genes expressed differently (25, 26). 

In our recent pilot study, we examined the effects of different dosages of E2 (0.3, 0.7, and 1 nmol) in combination with P4 (63.5 nmol) on the proliferation and survival rate of human endometrial stromal cells. Our data showed that 0.3 nmol of E2 with 63.5 nmol of P4 had a significantly higher proliferation rate than the other examined dosages of E2. By using 0.3 nmol of E2 with 63.5 nmol of P4 in another part of this experiment, our molecular observation demonstrated that despite any significant difference in expression of *LIFR* and *IL-1R*, the level of *αV* and *ß3* integrin expressions significantly increased (27). However, the interaction of these steroidal hormone-treated cells with the embryo was unclear and should be evaluated. Because of the limited availability of human embryos, a number of studies used surrogate embryos in designing implantation models. A few studies employed mouse blastocysts, while most were conducted with trophoblast spheroids derived from cell lines (20). 

According to the role of implantation models to facilitate evaluation of the implantation process, the present study aimed to determine the effects of E2 (0.3 nmol) and P4 (63.5 nmol) on the interaction between mouse embryo and human endometrial mesenchymal cells, and the gene expressions related to implantation (*αV* and *ß3* integrins, *IL-1R*, and *LIFR*) using a two-dimensional model.

## Materials and Methods

Reagents and materials of this research were obtained from Sigma Aldrich (Munich, Germany), unless mentioned otherwise. 

### Human endometrial samples

The Ethics Committee of the Medical Faculty of Tarbiat Modares University (no. 1394.137) approved this experimental study. Written informed consent was taken from all patients. The endometrial samples were obtained from healthy fertile women aged 25-35 years (n=10) during the proliferative phase who underwent hysteroscopy for non-pathological conditions. The patients did not have any exogenous hormone treatment for 3 months before the surgery. The normal morphology and normal menstrual cycle of the endometrial tissue was proven by histological examination and confirmed by an experienced histopathologist.

### Cell isolation and culture

The tissues were washed in phosphate-buffered saline (PBS), cut into small 1 mm pieces in Dulbecco’s modified Eagle’s medium/Hams F-12 (DMEM/F-12, Invitrogen, UK) that contained 100 mg/ml penicillin G sodium, 100 mg/ml streptomycin sulfate B, and 10% fetal bovine serum (FBS, Invitrogen, UK). The tissues were then subjected to mild enzymatic digestion according to a method by Chan et al. (28). Collagenase type 1 (300 µg/ ml) and deoxyribonuclease type I (40 µg/ml) were used to digest the tissue fragments into single cells along with the mechanical methods. In order to remove glandular and epithelial components, the resulting suspension were passed through 100 and 40 sieve meshes (Becton Dickinson, USA). Finally, endometrial stromal cells were cultured to the fourth passage using DMEM/F-12 that contained antibiotics and 10% FBS, and incubated at 37°C in 5% CO_2_.

### Flow cytometric analysis of endometrial cells 

After the fourth passage, we confirmed the immunophenotype of the endometrial cells using flow cytometric analysis to evaluate mesenchymal (CD90, CD73, and CD44) and hematopoietic markers (CD45 and CD34). A total of 1×10^5^ endometrial cells were suspended in 50 µl of PBS and incubated with direct fluorescein isothiocyanate (FITC)-conjugated antibodies (anti-human CD90, CD44, and CD45, 1:50 dilutions) and direct phycoerythrin (PE)-conjugated antibodies (antihuman CD73 and CD34; 1:50 dilutions) at 4°C for 45 minutes. Finally, 200 µl of PBS was added and the cells were examined with a FACSCalibur apparatus (Becton Dickinson, USA).

### Preparation of the media and cell culture

After the fourth passage, the mesenchymal stromal cells were collected and divided into two groups, experimental and control. The cells were cultured in the presence of 0.3 nmol E2 and 63.5 nmol P4 (27) (Aburaihan, Iran) in the experimental group. The cells were cultured in the absence of any hormone treatment in the control group. 

In order to prepare an initial concentration, E2 and P4 were dissolved in 100% ethanol and then suspended in media that contained 10% FBS to achieve a final working concentration (29, 30). The media that contained the hormones was allowed to incubate overnight in order to evaporate the ethanol. In each group, endometrial mesenchymal stromal cells were cultured in 48-well (15×10^3^ cells per well) plates using DMEM/F-12 that contained antibiotics and 10% FBS for 5 days. On the fifth day of culture, these cells were co-cultured with mouse embryos at the blastocyst stage.

### Superovulation and blastocyst collection

Adult female (8-10 weeks old, n=25) and male (8-12 weeks old, n=10) National Medical Research Institute (NMRI) mice were used in this study. The mice were housed under 12 hour light/12 hour dark conditions at 20-25°C with enough humidity, water and food in the laboratory animals house at Tarbiat Modares University (Iran).

The adult female mice were superovulated with an intraperitoneal injection of 7.5 IU pregnant mare serum gonadotropin (PMSG, Folligon, Intervet, Australia) followed by an intraperitoneal injection of 10 IU human chorionic gonadotropin hormone (hCG, Choragon, Germany) 48 hours later. Then, the mice were individually mated with fertile males. Normal morphology blastocyst embryos were collected from the uterine horns and transferred on the cultured endometrial mesenchymalstromal cells in both groups (3 embryos per well and 3 wells per group) for a period of 48 hours.

### Inverted microscope

During culture period and after embryo transfer, the endometrial mesenchymal stromal cell proliferation and implantation process was followed by inverted microscope assessments every 12 hours in both groups. 

### Scanning electron microscopy

The samples in the experimental and control groups were examined by scanning electron microscopy (SEM) for ultrastructural assessment of embryo implantation. The specimens (3 embryos per well and 3 wells per group) were fixed in two steps of 2.5% glutaraldehyde in PBS and 1% osmium tetroxide in the same buffer for 2 hours, respectively. After dehydration with ethanol, the specimens were dried, mounted, and coated with gold particles (Bal-Tec, Switzerland), and examined by SEM (Philips XL30, Netherland).

### RNA isolation and reverse transcription reaction

RNA was isolated from endometrial mesenchymal stromal cells after co-culture with embryos in each group of 3 embryos per well and 3 wells per group using the RNeasy Mini Kit (Qiagen, Germany). The RNA samples were treated with DNase to eliminate any genomic DNA contamination just prior to cDNA synthesis. The RNA concentration was determined by spectrophotometry. Then, the cDNA was synthesized in a total volume of 20 µl using a cDNA kit (Fermentas, EU) and stored at -80°C until use. All experiments were repeated three times.

### Quantitative real-time reverse transcription- polymerase chain reaction assays 

The primers for real time reverse transcription- polymerase chain reaction (RT-PCR) were newly designed using GenBank (http://www.ncbi.nlm.nih.gov) and synthesized at CinnaGen Company (Iran) (Table 1). The housekeeping gene (*ß-actin*) was used as an internal control. After cDNA synthesis, we performed real time RT-PCR with an Applied Biosystems real-time thermal cycler according to the QuantiTect SYBR Green RTPCR kit (Applied Biosystems, UK). For each sample, the reference gene and the target genes (*αV* and *ß3* integrins, *IL-1R*, and *LIFR*) were amplified in the same run and melting curve analysis was used to confirm the amplified product. The real-time thermal condition included a holding step: 95°C 10 minutes and cycling step: 95°C 15 seconds, 60°C 1 minute was continued by a melting curve step: 95°C 15 seconds, 60°C 1 minutes and 95°C 15 seconds . The relative quantification of target genes was determined using the Pfaffl method (31). All experiments were repeated three times. 

### Statistical analysis 

Statistical analysis was performed with SPSS version 22.0 software. Quantitative variables were expressedas mean ± SD. The results of real-time RT-PCR were compared by the independent samples t test. P=0.05 were considered statistically significant. 

## Results

### Flow cytometric analysis

**Fig.1:**
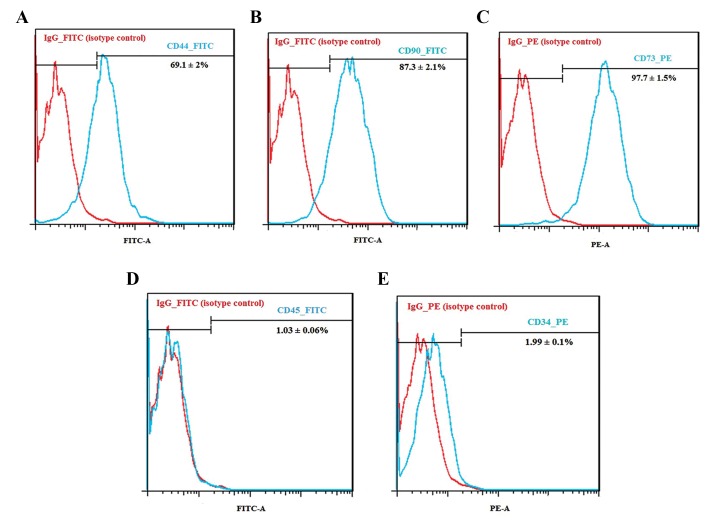
Flow cytometry analysis of passage-4 cultured endometrial stromal cells. The percentages of cells with different markers were demonstrated as A. CD44, B. CD90, C. CD73, D. CD45, and E. CD34. Analysis showed that the cultured endometrial cells stained negative for CD45 (D) and CD34 (E). Diagramsof red and blue are related to isotype control and test samples, respectively. Each diagram is representative of three independent experiments.

Immunophenotype of cultured endometrial cells after the fourth passage showed the following: 1.5% ± 97.7 (CD73), 87.3 ± 2.1% (CD90), 69.1 ± 2% (CD44), 1.99 ± 0.1% (CD34), and 1.03 ± 0.06% (CD45, Fig .1). 

**Table 1:** Characteristics of primers used for the real-time reverse transcription-polymerase chain reaction assay


Target gene	Primer pair sequences (5´-3´)	Accession number	Fragment size (bp)	T (˚C)

*αV*	ATCTCAGAGGTGGAAACAGGA	NM_002210.4	21	58.09
	TGGAGCATACTCAACAGTCTTTG		23	58.68
*β3*	AGTAACCTGCGGATTGGCTTC	NM_000212.2	21	60.68
	GTCACCTCGTCAGTTAGCGT		20	59.76
*LIFR*	TGTAACGACAGGGGTTCAGT	NM_001127671.1	20	58.58
	GAGTTGTGTTGTGGGTCACTAA		22	58.46
*IL-1R*	GGCACACCCTTATCCACCAT	NM_001261419.1	20	59.74
	GCGAAACCCACAGAGTTCTCA		21	60.54
*Β-actin*	TCAGAGCAAGAGAGGCATCC	NM_001101.3	20	60.5
	GGTCATCTTCTCACGGTTGG		20	60.5


*LIFR*; Leukemia inhibitory factor receptor and *IL-1R*; Interleukin-1 receptor.

### Morphological observation

**Fig.2:**
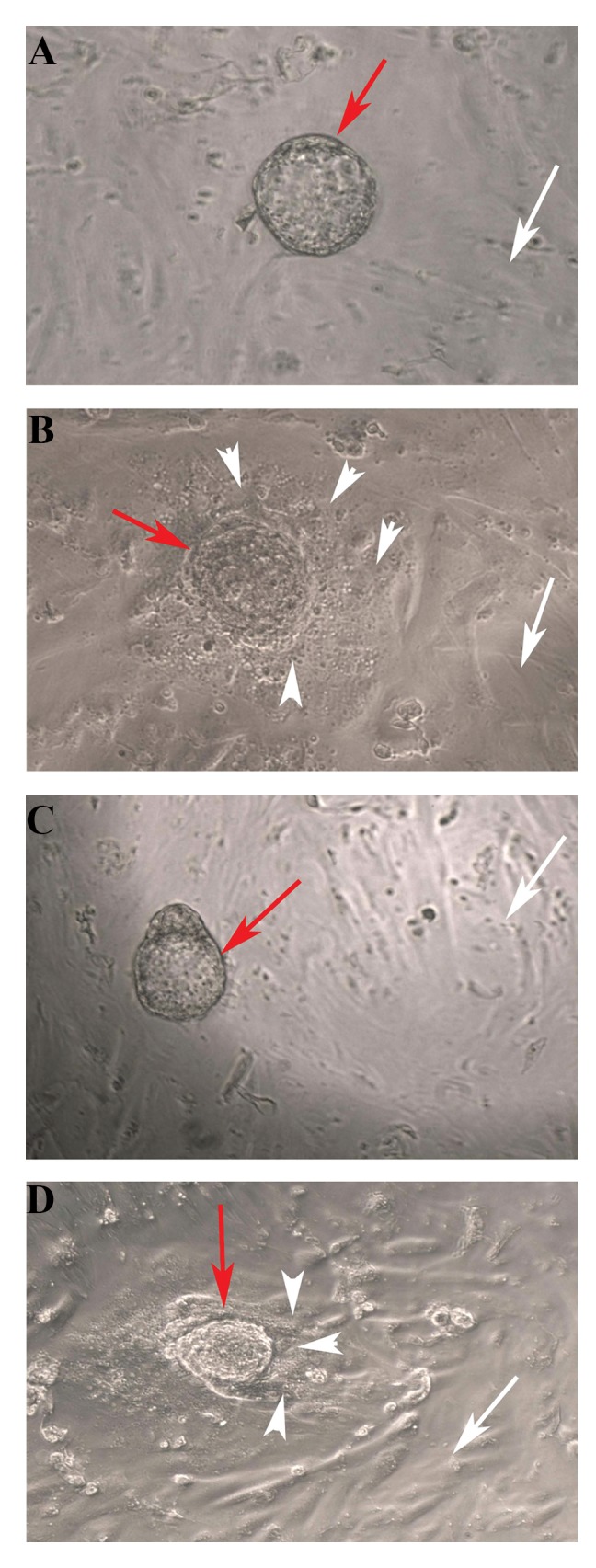
Phase-contrast imaging of mouse embryo co-cultured with human endometrial mesenchymal stromal cells. A, B. Control group (without steroid hormones), C, and D. Treated group with steroid hormones; 17β-estradiol (E2; 0.3 nmol) and progesterone (P4; 63.5 nmol) (scale bar: 100 μm). A, C. At 0 hours of co-culture. B, D. After 48 hours of co-culture. The red arrows show mouse blastocysts during the co-culture period. The white arrows show human endometrial mesenchymal stromal cells. The arrowhead show expanded trophoblastic cells.

The morphology of the co-cultured mouse embryos on the top of endometrial mesenchymal stromal cells as seen under an inverted microscope. The morphology in the two studied groups was similar and demonstrated in the Figure 2. The endometrial cells showed a flattened monolayer. As these micrographs indicated, the embryonic cells were spread on the endometrial mesenchymal stromal cell layer and attached tightly to these cells. The trophoblastic cells were outgrowth around the embryo. 

### Scanning electron microscopy 

**Fig.3:**
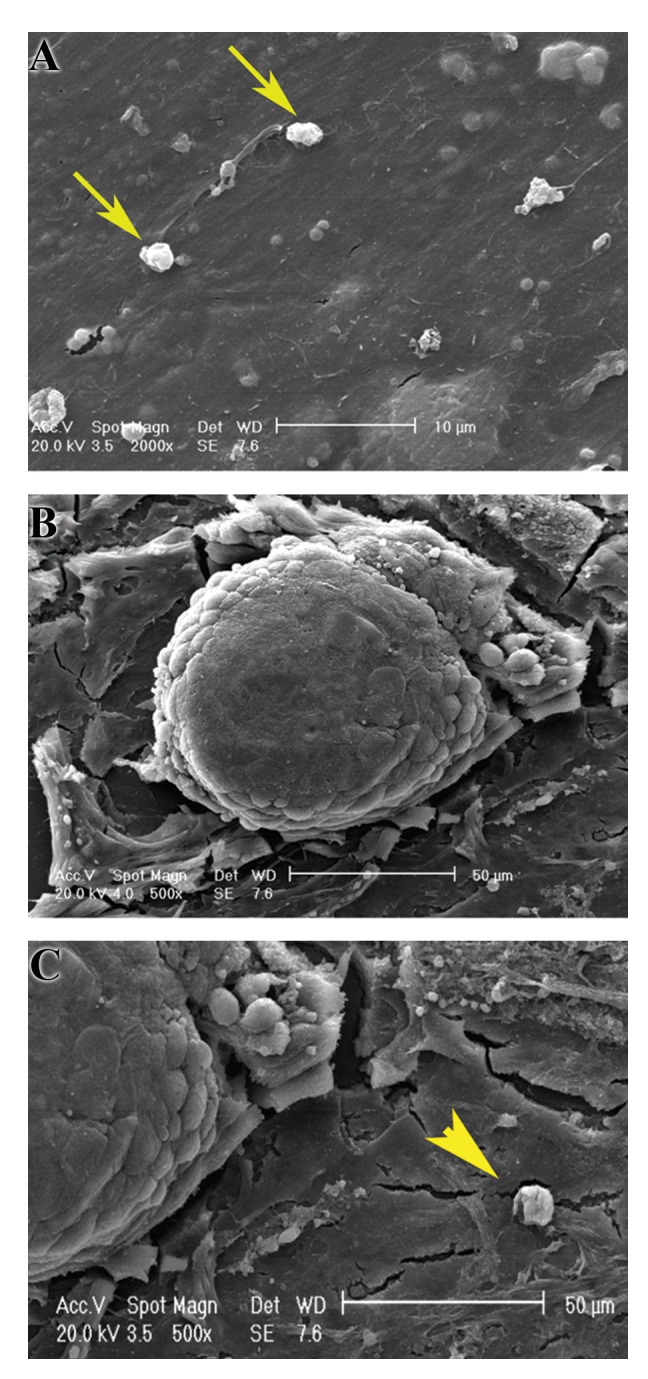
Scanning electron micrograph of mouse embryos co-cultured withhuman endometrial mesenchymal stromal cells. A. The arrows show some cell secretions on the apical surfaces of endometrial stromal cells, B. Mouse embryo, and C. The arrowhead shows pinopode-like structureon the apical surface of the endometrial cell.

The scanning electron micrographs of cultured endometrialmesenchymal stromal cells and mouse embryos were seenin the Figure 3A-C. The ultrastructural observations did notshow the prominent difference between the two groups. Themesenchymal stromal cells had a spindle shape and flattenedcells which attached to the floor of plate. In both groups, weobserved the presence of pinopodes-like structures (yellowarrowhead in Fig.3C) and cell secretions on the apical surfaces of endometrial mesenchymal stromal cells (yellow arrow in Fig.3A). 

### Real-time reverse transcription-polymerase chain reaction

**Fig.4:**
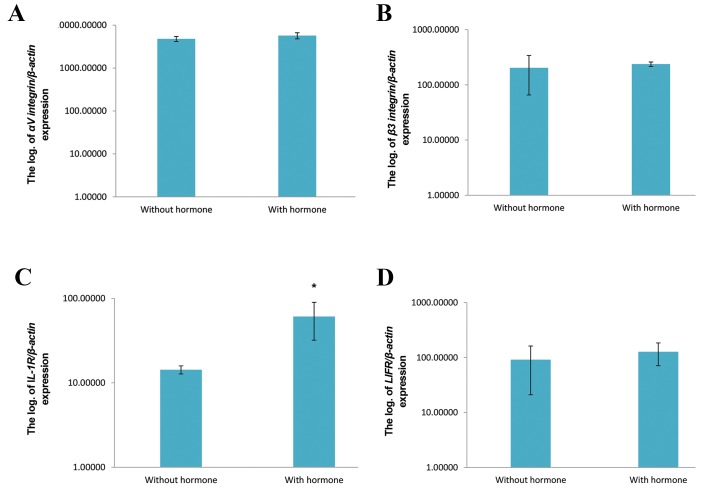
Comparison of gene expressions related to implantation to ß-actin in the treated and non-treated groups. . ; Significant difference with the control group; (P≤0.05).

At the molecular level, we noted the following ratio expressions of *αV* (5720.95 ± 929.09) and *ß3* (237.92 ± 22.18) integrins, and *IL-1R* (60.96 ± 28.96) and *LIFR* (127.59 ± 56.73) genes to the housekeeping gene in the experimental group. The ratio expressions in the control group were 4800.78 ± 646.85 (*αV* integrin), 203.61 ± 137.99 (*ß3* integrin), 14.29 ± 1.57 (*IL-1R*), and 91.62 ± 70.62 (*LIFR*). The expression of *IL-1R* significantly increased (P=0.05) in the experimental group compared to the control group. *αV* and *ß3* integrins, and *LIFR* gene expression did not differ in these groups (Fig .4).

## Discussion

In this study, we sought to improve an implantation model by using steroidal hormone-treated human stromal endometrial cells that followed our previous study. We have evaluated the interaction between mouse embryo and endometrial mesenchymal stromal cells under the influences of E2 and P4 at the morphological, ultrastructural, and molecular levels. For embryo implantation, alterations in the structure and function of endometrial cells are critical. 

Our observations have shown some signs of receptive endometrial characteristics on the apical surfaces of the endometrial mesenchymal stromal cells such as cell secretions and the presence of the pinopode-like structures. It has been determined that the steroidal hormones play an important role in embryo implantation (24). However, our observations did not show any obvious morphological and ultrastructural differences between the steroid hormone treated group to the non-treated group. These observations might be related to the insufficient dosage of hormones used in this study. It has been shown that the effects of steroid hormones are mainly dose-dependent which agrees with this suggestion (4). More studies would be necessary to confirm this suggestion. On the other hand, the secreted factors by embryo impact the differentiation and preparation of endometrial mesenchymal stromal cells for attachment to the embryo. However, more studies need to prove this suggestion. 

In the current study, we performed quantitative analysis to detect ultrastructural changes. In order to better evaluate the effects of these hormones, additional experiments would be required. Evidences exist that expression of pinopodes and other ultrastructural changes in the endometrial cells are hormone dose-dependent (4). Probably the dosages of E2 and P4 used in this study were not adequate to show remarkable ultrastructural changes. Stavreus-Evers et al. (32) reported the importance of increased P4 serum levels of P4 in pinopode development. An association existed between formation of pinopodes to the concentrations of P4 in the human endometrium. Ma et al. observed that estrogen at different physiological concentrations could initiate implantation of an embryo but the implantation window remained open for an extended period at lower estrogen levels and rapidly closed at higher E2 levels (33).

In the current study, for the first time, we evaluated the expression of some genes related to implantation in the presence of steroid hormones. Our molecular analysis showed that despite an increase in *IL-1R* expression in the hormone treated group compared to the control, the pattern of other genes (*αV, ß3* integrins, and *LIFR*) did not differ in these two groups. These observations differed from our previous experiment (27). We emphasized that these two studies had a similar design, except for the presence of embryos in the present study.

The aim of the present study was to examine the effect of an embryo co-culture with these hormone-treated cells. Thus it could be concluded that these different expression pattern of genes related to implantation might be due to the presence of the embryos. The trophectoderm of an embryo is the main source of P4 and a number of other hormones that could be secreted thus it could change the level and balance of hormones within the media. In agreement with this suggestion, some reports indicated that E2 and P4 differently modulate the expression of genes related to the implantation in a dose-dependent manner (34-36). Horcajadas et al. (36), in an in vivo study, assessed expressions of four genes in the human endometrium under the influence of E2. They observed that during the implantation window only three genes upregulated (*osteopontin, apolipoprotein D, Dickkopf*) and one downregulated (*olfactomedin-1*). 

Dassen et al. (37), with an in vitro culture of a human endometrial explant in the presence of E2 and P4, reported that the expression of some genes associated with embryo implantation such as IL1RL1 and CRABP2 depended on the duration of E2 exposure.

Defects in the expression of genes related to implantation result in implantation failure during the receptive phase by changing the dosage of hormones or lack of steroidal hormone signaling (33, 38).

According to the best of our knowledge, limited studies have evaluated the expression of genes related to implantation in the in vitro model. The results are influenced by the use of different assay methods, the use of different protocols for sample preparation, differences between species, and the manner of steroid usage.
